# Intelligent automatic sleep staging model based on CNN and LSTM

**DOI:** 10.3389/fpubh.2022.946833

**Published:** 2022-07-27

**Authors:** Lan Zhuang, Minhui Dai, Yi Zhou, Lingyu Sun

**Affiliations:** ^1^Staff Hospital, Central South University, Changsha, China; ^2^Teaching and Research Section of Clinical Nursing, Xiangya Hospital of Central South University, Changsha, China; ^3^Department of Ophthalmology, Xiangya Hospital, Central South University, Changsha, China

**Keywords:** EEG signal, convolutional neural network, long-term and short-term memory, sleep stage, multichannel, feature fusion

## Abstract

Since electroencephalogram (EEG) is a significant basis to treat and diagnose somnipathy, sleep electroencephalogram automatic staging methods play important role in the treatment and diagnosis of sleep disorders. Due to the characteristics of weak signals, EEG needs accurate and efficient algorithms to extract feature information before applying it in the sleep stages. Conventional feature extraction methods have low efficiency and are difficult to meet the time validity of fast staging. In addition, it can easily lead to the omission of key features owing to insufficient a priori knowledge. Deep learning networks, such as convolutional neural networks (CNNs), have powerful processing capabilities in data analysis and data mining. In this study, a deep learning network is introduced into the study of the sleep stage. In this study, the feature fusion method is presented, and long-term and short-term memory (LSTM) is selected as the classification network to improve the accuracy of sleep stage recognition. First, based on EEG and deep learning network, an automatic sleep phase method based on a multi-channel EGG is proposed. Second, CNN-LSTM is used to monitor EEG and EOG samples during sleep. In addition, without any signal preprocessing or feature extraction, data expansion (DA) can be realized for unbalanced data, and special data and non-general data can be deleted. Finally, the MIT-BIH dataset is used to train and evaluate the proposed model. The experimental results show that the EEG-based sleep phase method proposed in this paper provides an effective method for the diagnosis and treatment of sleep disorders, and hence has a practical application value.

## Introduction

People spend one-third of their time sleeping every day, and sleep is an important and necessary physiological process of the human body. Sleep quality will seriously affect people's physical and mental health. The acceleration of the pace of life in modern society and the increase of life pressure have also affected people's sleep quality, causing many people to suffer from sleep diseases (1). Clinical medicine analyzes and judges sleep quality by monitoring patients' sleep status, so as to achieve the purpose of treating or preventing sleep diseases. The collection of sleep data is the basis of sleep quality analysis. At present, the research on automatic sleep staging has made some progress. The sleep staging process requires professionals to receive a lot of training and accumulate on-site experience, which is a complex and cumbersome process. Therefore, it is necessary to combine sleep with artificial intelligence to automatically and effectively evaluate sleep stages. Sleep is the most important physiological process related to human health. With the acceleration of people's pace of life and the increase in stress, the incidence rate of sleep disorders is also increasing. At present, patients tend to be younger. Therefore, the research on sleep has attracted extensive attention from scholars. From a clinical point of view, sleep staging is an important basis for monitoring sleep quality and detecting sleep-related diseases. Nowadays, people all over the world have difficulty sleeping or even insomnia, and more and more diseases are caused by sleep problems. Studies have shown that sleep quality plays a very important role in the regeneration of memory neurons. Poor sleep quality or insufficient sleep will damage memory to some extent. In addition, some studies have proved that sleep disorder has been identified as one of the earliest potential biomarkers for the progression of Alzheimer's disease, and monitoring sleep changes is helpful for early intervention of dementia (2). Therefore, how to correctly stage sleep is an important basis for monitoring sleep quality and diagnosing sleep-related diseases. It has important practical significance and application value to improve sleep quality or diagnose sleep disorders through artificial intelligence. The convolutional neural network is a feedforward neural network, including an input layer, an output layer, convolution layer, and pool layer (3). Its special network structure can effectively reduce the complexity of the neural network. It has achieved great success in many fields, particularly in image recognition. At present, CNN has been successfully applied to many specific projects, such as original EEG data, target recognition, and handwritten character recognition. It also occupies a place in many fields, such as analyzing visual images, speech recognition, and predicting human traffic (4). As we all know, CNN has the characteristics of shared weight structure and transformation invariance. CNN consists of input and output layers and multiple hidden layers. The hidden layer can be a convolution layer, a pooling layer, or a full connection layer. Compared with other traditional algorithms, CNN uses relatively less preprocessing. This means that CNN can learn from the manually designed filter in the traditional algorithm. Its main advantage is that it can replace prior knowledge and artificial design feature engineering (5). Therefore, this study aims to study a new automatic sleep classification method based on EEG signals, including signal preprocessing, feature extraction, and classification and recognition. The main research content of this paper is to realize the automatic segmentation of sleep by using signal processing method and pattern recognition theory, and establish an automatic sleep staging system with high accuracy and fast calculation speed to provide more effective and convenient sleep staging methods for medical researchers, and reduce the workload of clinical diagnosis.

The specific contributions of this study include the following:

(1) Based on EEG signals and a deep learning network, this paper proposes an automatic sleep staging method based on multi-channel EEG, and CNN-LSTM is used for supervised learning of sleep staging on original EEG and EOG samples.
(2) The EEG signals are filtered and extracted, respectively, and the features are extracted by CNN and LSTM in parallel. The two features are fused for classification.
(3) By introducing an electro-oculogram and fusing the modal information between EEG and EOG, the classification accuracy of each stage is improved.
(4) When decomposing EEG signal and using the method proposed in this paper and the characteristics of rhythm wave, relative energy, and complexity for automatic sleep staging, by using the information complementarity between different modes, the calculation time of feature extraction can be reduced, the error can be reduced, and the accuracy of automatic staging can be improved.

The remainder of this paper is organized as follows. Section Related work discusses related work, followed by the composition of brain waves in EEG and signal detection in Section EEG signal. Sleep staging criteria and specific sleep stages are discussed in Section Sleep stage. Section CNN-LSTM sleep stages algorithms presents CNN-LSTM sleep stages algorithms, Section Contrast experiment and result analysis details the contrast experiment and result analysis, and Section Conclusion and future work provides a conclusion with a summary and future research direction.

## Related work

Sleep experts can determine the quality of sleep by recording electrical activity through sensors connected to different parts of the body. A set of signals collected by these sensors is called polysomnography (PSG). A series of physiological signals were recorded, including EEG, EOG, EMG, and ECG. EEG signals often contain a lot of information about physiological activities (6). It is an extremely complex bioelectrical signal. It provides important analytical reference information for neurology, medicine, and other disciplines. It can accurately reflect the activity of the brain and is gradually widely used in sleep research. Each person has different characteristics and amplitudes of EEG signals in different sleep states, which reflects the different and complex functions of the brain in different stages (7). The classification of different characteristics of different sleep stages is called sleep stage. It is not only the standard for judging sleep quality, but also the basis for diagnosing some brain diseases. It has important clinical significance and broad application prospects for the treatment of sleep disorders (8). The early classification standard of sleep stage is the R&K standard, which was proposed by Rechtchaffen and Kales in 1968. The human sleep process is divided into three types: wake, non-rapid eye movement sleep (NREM), and rapid eye movement sleep (REM) (9). According to expert standards, polysomnography (PSG) is divided into stages every 30 s. Professional sleep data evaluation can only be carried out in the hospital, and then experts judge each paragraph according to the classification criteria through visual observation. It is very cumbersome, time-consuming, and prone to subjective errors (10). An 8-h PSG data requires professional doctors to spend 2–4 h on sleep staging, which is very inefficient. Therefore, the development of automatic sleep staging is of great significance. Automatic staging is the research of feature extraction and classification of EEG data collected by using signal processing, pattern recognition, and computer technology. Compared with manual staging, it saves time and effort. It can not only improve the efficiency of data judgment and classification, but also reduce the misjudgment caused by doctors' subjective factors in the process of staging (11, 12).

In order to avoid the misjudgment of subjective consciousness caused by the manual recognition of the sleep stage by experts, more and more scholars are committed to the research of automatic sleep staging. Sleep data staging belongs to the category of pattern recognition (13). In recent years, with the rapid development of pattern recognition methods, a large number of classification methods have been found and used, accompanied by continuous changes and improvements. Common recognition methods are as follows: Decision Tree (DT), Artificial Neural Networks (ANNs), Linear Discriminant Machine (LDM), and Support Vector Machine (SVM) (14, 15). An interpretable machine learning algorithm was used to evaluate the interrater reliability (IRR) of sleep stage annotation among sleep centers by Liu et al. (16). Intracenter and intercenter assessments were conducted on 679 patients without sleep apnea from six sleep centers in China's Taiwan. Bandyopadhyay and Goldstein (17) provided a concise overview of relevant terminology, definitions, and use cases of AI in sleep medicine. They conclude that artificial intelligence is a powerful tool in healthcare that may improve patient care, enhance diagnostic abilities, and augment the management of sleep disorders. Bozkurt et al. (18) proposed a new approach to sleep staging, which is one of the diagnostic procedures for the disease. An artificial intelligence-based sleep/awake system detection was developed for sleep staging processing. Photoplethysmography (PPG) signal and heart rate variable (HRV) were used in the study. Zhu et al. (19) adapted edge AI to develop a lightweight automatic sleep staging method for children using single-channel EEG. The trained sleep staging model will be deployed to edge smart devices so that the sleep staging can be implemented on edge devices which will greatly save network resources and improve the performance and privacy of the sleep staging application. Cesari et al. (1) conducted this study to evaluate interrater reliability between manual sleep stage scoring performed in two European sleep centers and automatic sleep stage scoring performed by the previously validated artificial intelligence-based Stanford-STAGES algorithm. Abbasi et al. (20) proposed an internet of things and ensemble-based automatic sleep stage classification in this study. Twelve EEG features, from nine bipolar channels, were used to train and test the base classifiers, including a convolutional neural network, support vector machine, and multilayer perceptron. Eldele et al. (21) proposed a novel attention-based deep learning architecture called AttnSleep to classify sleep stages using single-channel EEG signals. This architecture started with the feature extraction module based on a multi-resolution convolutional neural network (MRCNN) and adaptive feature recalibration (AFR). Prochazka et al. (22) devoted to the analysis of multi-channel biomedical signals acquired in the sleep laboratory. The goal of the study was to visualize the features associated with sleep stages as specified by an experienced neurologist in their adaptive classification. Delimayanti et al. (23) demonstrated the utilization of features extracted from EEG signals *via* FFT to improve the performance of automated sleep stage classification through machine learning methods. Gao et al. (24) developed a novel program called GI-SleepNet, a generative adversarial network (GAN)-assisted image-based sleep staging for mice that is accurate, versatile, compact, and easy to use. Loh et al. (25) proposed a deep learning model based on a one-dimensional convolutional neural network (1D-CNN) for CAP detection and homogenous 3-class classification for sleep stages: wakefulness (W), rapid eye movement (REM), and NREM sleep. The proposed model was developed using standardized EEG recordings. Manzano et al. (26) presented the first approximation using a single channel and information from the current epoch to perform the classification. The complete Physionet database has been used in the experiments. The main research objective of this paper is to realize the research on EEG sleep automatic staging by using signal processing method and deep learning theory, and establish an automatic sleep staging system with high accuracy and fast calculation speed to provide a more effective and convenient sleep staging methods for medical researchers, reduce the workload of clinical diagnosis, and reduce the error rate.

## EEG signal

The brain is mainly responsible for controlling and regulating the thinking and conscious activities of the human body. EEG is a random signal reflecting the electrical activities of brain tissue. The cerebral cortex of the brain is the highest center of the nervous system regulating body movement, which is responsible for the cognitive and emotional functions of the brain. Various human activities and senses can find corresponding regions in the cerebral cortex, and each region has its specific role and function. Neuron cells in the cerebral cortex are the reason why human beings have complex activities as advanced organisms. The condition for complex activities is that each neuron cell can communicate through dendritic connections, which makes human activities have countless possibilities (27).

Scientific research shows that EEG signal is formed by a large number of neuronal cells. It is a non-linear mixture of multiple cells connected by non-linear coupling. From the perspective of chaos theory, the human brain is a complex non-linear dynamic system. EEG signal has the following characteristics: weak signal and strong noise; high complexity of signal components; prominent frequency characteristics; non-linearity; and non-stationarity.

### Composition of brain waves

The root cause of EEG is the response of neurons in the cerebral cortex to electrical activity. The signals generated by the potential activity of nerve cells can be obtained by placing electrodes on the scalp. The amplitude, frequency, and phase of the waveform contained in the EEG have certain characteristics. The bandwidth of EEG is 0.5–100 Hz, and the 0.5–30 Hz portion of spontaneous EEG is considered in clinical medicine. According to the frequency characteristics, it can be divided into four basic rhythm waves and non-basic waves. The regular waves in the sleep process are rhythm waves, and the irregular waves are non-basic waves. There are four kinds of rhythm waves:

(1) δ wave: 0.5–4 Hz, amplitude 20–200 μV. δ wave is the wave with the lowest frequency in the brain, which is obvious in the forehead. It appears more often in the EEG of infants or people with intellectual disabilities, and it will not occur in normal people when they are awake; however, this wave can be produced when adults are in extreme fatigue, deep sleep, and deep anesthesia. When the cortex is inhibited, δ rhythmic waves appear.
(2) θ wave: 4–8 Hz, amplitude about 20–150 μV. θ wave is a low-frequency rhythmic wave in the human brain. It is the main EEG activity in teenagers aged 10–17 years. It is obvious in temporal and parietal areas. Similar to δ wave, it occurs only when the brain nerve is in a sleepy, depressed, or frustrated state, and it does not occur when normal people are awake, mtf(Modulation Transfer Function) wave, if someone is awake, the signal contains a lot of θ wave, should be paid attention to, its brain may have lesions, which will lead to disease.
(3) α wave: 8–13 Hz, amplitude <100 μV. α wave is the most obvious waveform with a high frequency in adult EEG. It has a stable existence in the state of closed and relaxed eyes, but it disappears immediately after being stimulated by the outside world. The α wave represents human logical reasoning and creative thinking, which are not available to other animals. The α wave is a basic waveform of health and an important rhythmic wave reflecting various indexes of the brain.
(4) β wave: 13–30 Hz amplitude of 5–20 V. The frequency is 13–30 Hz. β wave is in the range of 20–30 Hz β two waves. β wave is the fastest rhythmic wave in the brain, which is mainly concentrated in the frontal and central regions. β waves are the manifestation of the excited state of the cerebral cortex and can also be used as the characteristics of brain awakening. It can be detected when people are moving, thinking, reading, or watching movies.

There are three types of common non-fundamental waves as follows:

(1) K-complex wave: there is no exact frequency range, and is composed of a positive and negative sharp waves. It first appears negative and then positive, and the transient time is >0.5 s. The K-complex wave usually appears together with the spindle wave, which are fused together to determine the S2 phase. The K-complex wave is obvious in the top and central regions.
(2) Sawtooth wave: the frequency range is 2–7 Hz. The waveform is like a sawtooth shape. Its existence generally represents the beginning of a dream, that is, sawtooth waves generally exist when the human body is in a light sleep state or dreaming.
(3) Spindle wave: the frequency is 12–14 Hz, and the amplitude is the largest and constantly changing in EEG. At first, it is the lowest, the highest in the center, and then gradually decreases to the lowest. It is obvious in the parietal and midline regions of the brain. The spindle wave is the main characteristic waveform during light sleep.

### EEG detection

The EEG signal detection and recording equipment are composed of amplifier, electrode, display, etc. Electrodes are used to measure brain epidermal potential. Special electrodes need to be selected to measure EEG. In fact, the electrodes that can be used to measure brain potential include a tubular electrode, deep electrodes, disk electrodes, and so on. Different electrodes are designed to meet the different noise signals generated in the acquisition process of EEG signals. The conditions for selecting electrodes are as follows: small drift can reduce the noise interference of EEG; the polarization potential is stable, indicating good reproducibility; small photoelectric effect ensures that the electrode is not affected by light and is not easy to aging; and good mechanical properties mean long electrode life. Considering the above factors, the disk electrode is the most commonly used electrode for EEG measurement.

## Sleep stage

### Sleep staging criteria

**Figure 1:**
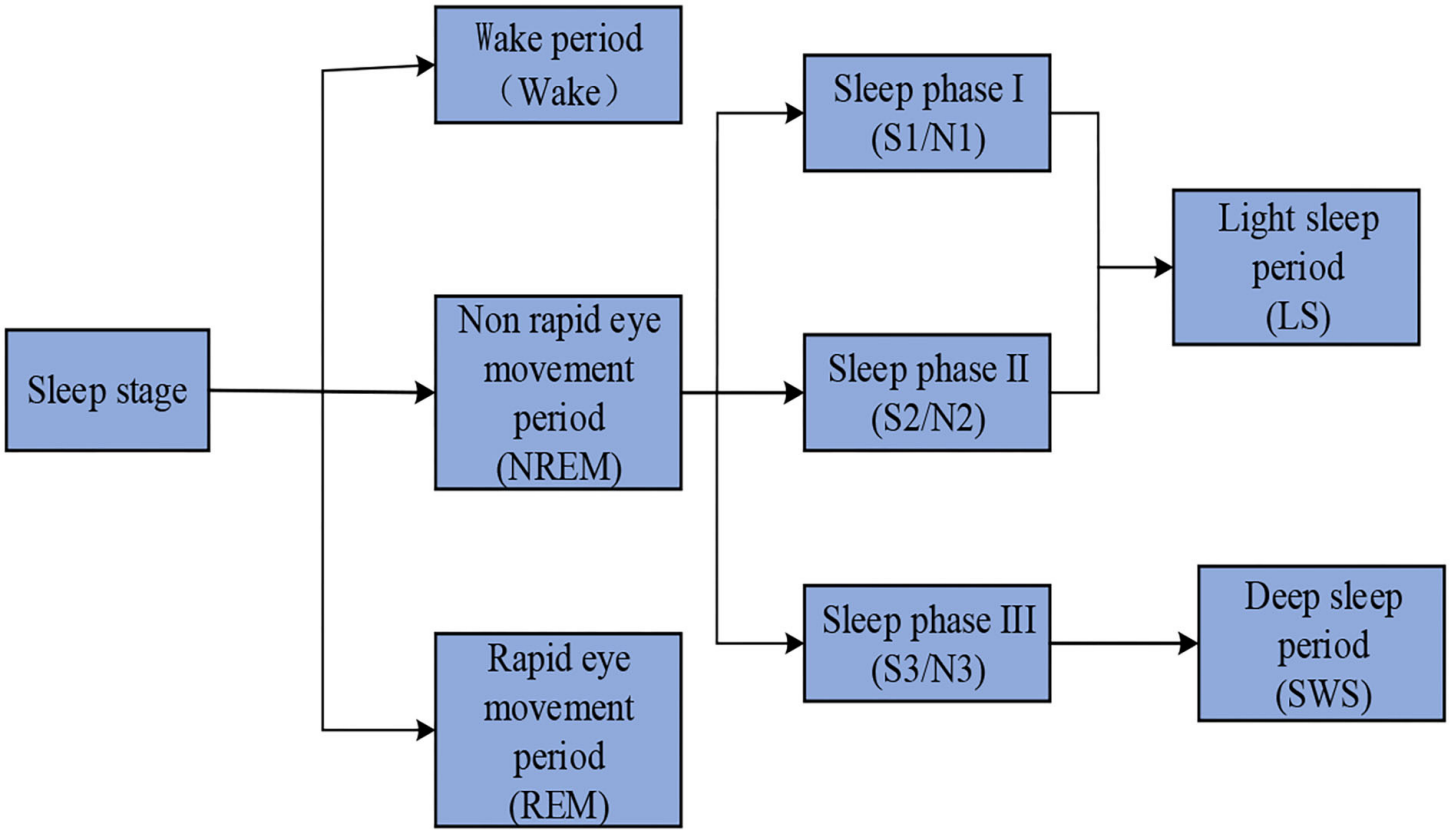
AASM sleep staging criteria.

The EEG changes during sleep. According to different eye, EEG, and EMG signals, a sleep grading system can be designed to respond to different sleep levels. The R&K staging standard proposed in the 1960s is widely used in the world. Sleep is divided into six periods: wake, non-rapid eye movement (NREM, including I, II, III, and IV), and rapid eye movement (REM). This standard is of great significance to study sleep staging, which was once called the “gold standard.” Later, researchers found that the characteristics of sleep stages III and IV were not obvious. Therefore, in 2007, the American Society of Sleep Medicine merged the two stages III and IV in the R&K standard, divided the total sleep period into five stages, and set the latest sleep staging standard (AASM standard), which is of epoch-making significance. The sleep staging standard selected in this paper is the AASM standard. Figure 1 presents the sleep staging criteria according to AASM.

### Specific introduction to sleep stages

According to the AASM standard, the sleep process is divided into five stages: waking stage (W), rapid eye movement stage (REM), light sleep phase I (N1), light sleep phase II (N2), and deep sleep phase (N3). Each sleep stage will be briefly discussed below.

Waking stage (W period): adults are generally awake for about two-thirds of the time in a day. At this stage, eye movement is obvious, which is the characteristic of frequent blinking. During the awake period, the EEG shows an uninterrupted alpha wave (frequency 8–13 Hz, amplitude 20–100 μV).

Light sleep phase I (N1): this phase often occurs when the awake state changes to other sleep states, or when there is more body movement during sleep, generally accounting for about 5–10% of the whole night's sleep. The duration of each occurrence is short, often only 1–7 min. The characteristics of this period are as follows: the content of the alpha wave decreases (<50%), and the content of the theta wave (frequency is 4–8 Hz and the amplitude is 20–150 μV) will slowly increase.

Light sleep phase II (N2): this phase occupies a longer time than light sleep phase I during the whole night, generally accounting for 44–55% of the sleep duration. This period is characterized by the gradual emergence of two new characteristics of EEG signals: sleep spindle wave and K-complex wave. These waveforms are most obvious in the EEG-type detection of the central electrode. At this stage, the θ waves of EEG signals increase and gradually appear at the same time as the δ wave (frequency is 0.15–4 Hz and amplitude is 20–200 μV).

Deep sleep period (N3): when people enter deep sleep, that is, when the sleep stage is in N3, the repair functions of the body and the visceral function are the strongest. At this stage, the cerebrospinal fluid in the brain will regularly clean the “biological waste” in the brain, which is very important for stabilizing mood, restoring energy, and adjusting mental state. This stage accounts for about 10–20% of the total sleep time. It is the deepest stage in the sleep process. The approximate frequency range of EEG signals at this stage is 0.5–2 Hz, and δ waves slowly increased, accounting for more than 20% of the waves.

Rapid eye movement (REM): this accounts for 20–50% of the total sleep process. The biggest feature of the REM phase is rapid eye movement, which is similar to the EEG signal of the N1 phase, and the REM sawtooth phase occurs. At this stage, α waves increased but showed an irregular pattern, and were accompanied by rapid eye movement.

## CNN-LSTM sleep stages algorithms

This paper will construct the automatic sleep stages model based on CNN and LSTM algorithms, train the model, and evaluate its classification performance through experiments.

### Deep neural networks

#### Convolutional neural network (CNN)

**Figure 2:**
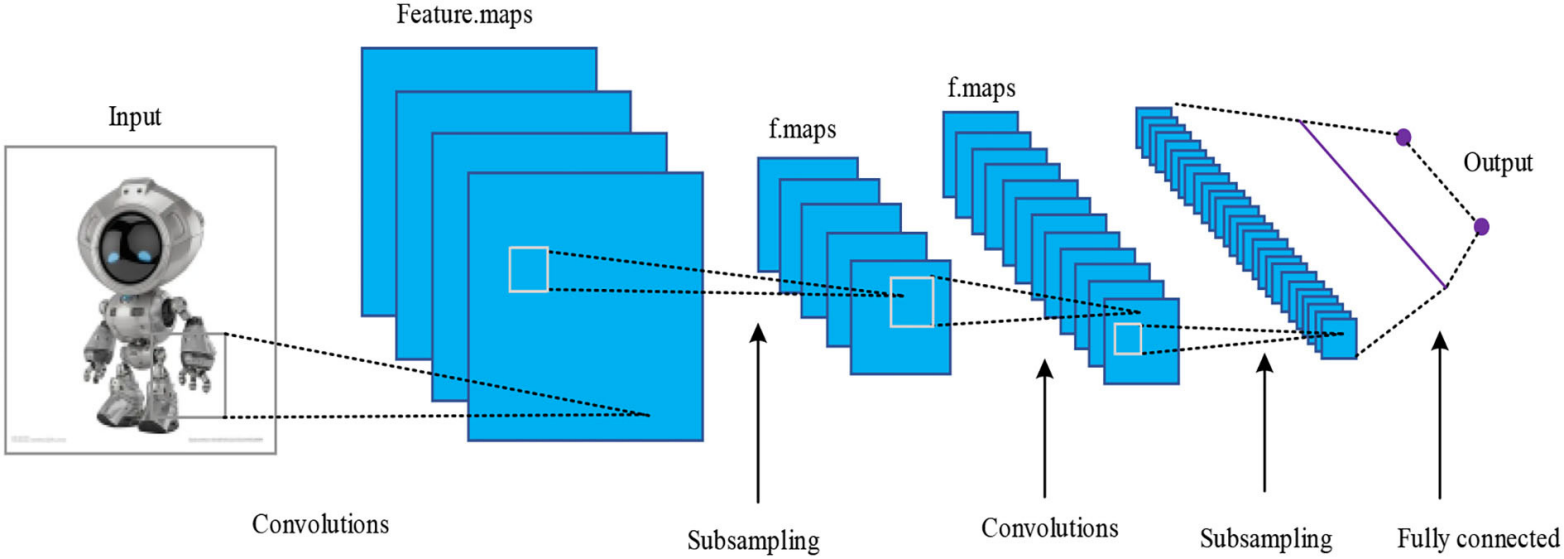
Typical CNN structure.

A convolutional neural network (CNN) is a feedforward neural network, and it is featured with weight sharing and local reception. A typical CNN structure is shown in Figure 2. The convolution neural network is constructed by imitating the visual perception mechanism of biology, which can carry out supervised learning and unsupervised learning. The sharing of convolution kernel parameters in the hidden layer and the sparsity of inter-layer connections enable the convolution neural network to learn grid-like topology features with less computation. The function of the convolution layer is to extract the features of the input data. It contains multiple convolution kernels. Each element of the convolution kernel corresponds to a weight coefficient and a bias vector, which is similar to a neuron of a feedforward neural network. Each neuron in the convolution layer is connected to multiple neurons in the area close to the previous layer, and the size of the area depends on the size of the convolution kernel.

In the structure, the output of the convolutional layer is locally connected to the input of the last layer, and it can be acquired by sliding the window gradually according to the step length. The corresponding local features with each parameter of the convolution kernel are multiplied and the weight of the kernel is kept unchanged, that is, weight sharing. Usually, in every convolutional layer, there are several convolution kernels and different features can be extracted. Through convolution, image features can be obtained, and after that, the classifiers can be trained according to these features directly, but such computation is huge and over-fitting occurs easily. Therefore, the addition of a pooling layer after the convolutional layer can shrink the feature map matrix and reduce the computation amount while enhancing the anti-jamming capability for feature identification, e.g., image deformation and distortion. Through local connection, weight sharing, and pooling, CNN greatly reduces the complexity of the network.

The convolutional neural network is frequently used in the processing of images and discrete signals, and its network structure mainly includes the input layer, the convolutional layer, the pooling layer, the full-connected layer, and the output layer.

(1) Convolutional layer

The convolutional layer is the most important layer in CNN, and it extracts efficient high-level feature information from the data of the input layer and inputs the regions divided by sliding windows, and conducts convolution operations with the kernel parameters. During each computation, the kernel window translates at the fixed step length in the input layer until the entire input participates in the convolution operation. Then with the activation function, add non-linear factors and construct the feature map. The output of the computation is also called feature mapping. Assume the input to be a two-dimensional tensor *I* and a two-dimensional convolution kernel *K*, CNN uses the cross-correlation function which is basically the same as the convolution operation and does not reserve the convolution kernel.


(1) $$\begin{array}{l} S\left(i,j\right)=\left(I\;*\;K\right)\left(i,\;j\right)\\ \;=\underset{m}{\sum }\underset{n}{\sum }I\left(m,n\right)K\left(i-m,j-n\right)\\ \;=\underset{m}{\sum }\underset{n}{\sum }I\left(i+m,j+n\right)K\left(m,n\right) \end{array}$$

In essence, CNN's convolution operation gets the local features from the weighted sum of local input through the convolution kernel and its convolution operation is as follows:

Convolution operation has strengths, such as sparse connection and parameter sharing. In the networks which connect all layers with a full-connected layer, each input and output cells are combined and connected, resulting in huge computation and easy over-fitting, exploding and vanishing gradient, and other issues. However, both the size and quantity of the convolution kernels are much fewer than the input nerve cell, and they can significantly reduce the number of weight parameters and have the characteristics of sparse weight. Besides, every weight factor in the convolution kernel has translated convolution operation with all neurons of the last layer, reducing the dense matrix operation in the full-connected layer and increasing the efficiency of reverse update of network parameters through weight and parameter sharing.

(2) Pooling layer

The pooling layer processes the output of the last layer through pooling functions, such as the maximum, mean, and *L*^2^ norm. It comprehensively takes into account the neighbor value of each position in the output of the last layer, conducts further feature localization processing on the original feature map, and maps it into another feature space. The pooling layer can reduce the scale of input, maintain the output of the features in the last layer, and effectively lower the dimensions of features so as to enhance the computational efficiency of the network.

(3) Full-connected layer

Full-connected layer maps the feature map output from previous layers into a feature vector and reduces the impact of spatial location on object identification. As the full-connected layer is based on dense matrix operation, it has low computational efficiency, and it is easy to contribute to over-fitting; therefore, Dropout is often used to increase the sparsity of the full-connected layer and reduce the feature redundancy among neurons.

#### LSTM network

A recurrent neural network (RNN) is a neural network that is suitable to be used in the processing of sequence data. RNN increases the lateral ties among cells, making it preserve the information state of the context windows when analyzing time sequence data, and it has been widely applied in natural language processing and machine vision which includes serial input. In practical applications, it mainly includes long short-term memory (LSTM) and gated recurrent cell (GRU).

**Figure 3:**
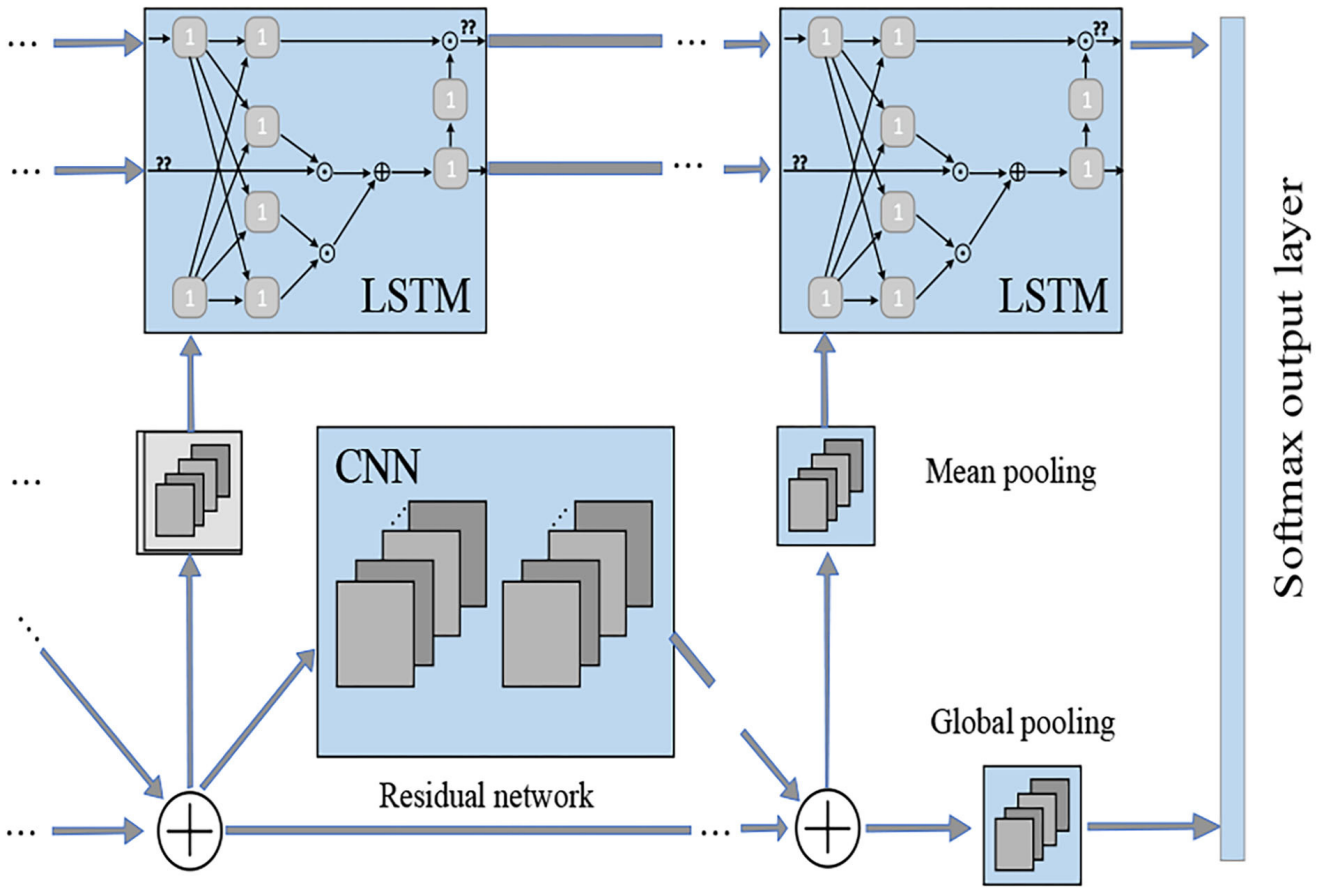
LSTM cell.

This paper mainly uses LSTM to acquire the time sequence features of sleep EEG. LSTM introduces logic control gates, and they are responsible for the memory and update of the history state. The input gate is responsible to control the impact of the input *x*_*t*_ on the current LSTM cell state *c*_*t*_, and the forgotten gate controls the impact of the state *c*_*t*−1_ of the last moment on *c*_*t*_. The cell structure of LSTM is shown in Figure 3.

The expressions among the LSTM forgotten door *f*_*t*_, input door *i*_*t*_,output door *o*_*t*_, and state vector ${\tilde{c}}_{t}$ within the cell can be given by:


(2) $$\begin{array}{rcl} f_{t}\; & = & \;\sigma \left(W_{f}\;\cdot \left[h_{t-1},x_{t}\right]\;+\;b_{f}\right) \end{array}$$

(3) $$\begin{array}{rcl} i_{t}\; & = & \;\sigma \left(W_{t}\cdot \left[h_{t-1},\;x_{t}\right]+b_{t}\right) \end{array}$$

(4) $$\begin{array}{rcl} {\tilde{c}}_{t}\; & = & \tanh\;\left(W_{c}\cdot \left[h_{t-1},\;x_{t}\right]\;+\;b_{c}\right) \end{array}$$

(5) $$\begin{array}{rcl} c_{t}\; & = & \;f_{t}\;\times \;c_{t-1}\;+\;i_{t}\;\times {\tilde{c}}_{t} \end{array}$$

(6) $$\begin{array}{rcl} o_{t}\; & = & \;\sigma \left(W_{o}\;\cdot \left[h_{t-1},\;x_{t}\right]+b_{o}\right) \end{array}$$

(7) $$\begin{array}{rcl} h_{t}\; & = & \;o_{t}\;\times \tanh\left(c_{t}\right) \end{array}$$

where *W*_•_ is the weight matrix, *b*_•_ is the bias vector, σ is logistic sigmoid function, and tanh is the hyperbolic tangent mapping activation function.

### Building of automatic sleep stages model based on neural network

#### CNN-LSTM automatic sleep stages model

**Figure 4:**
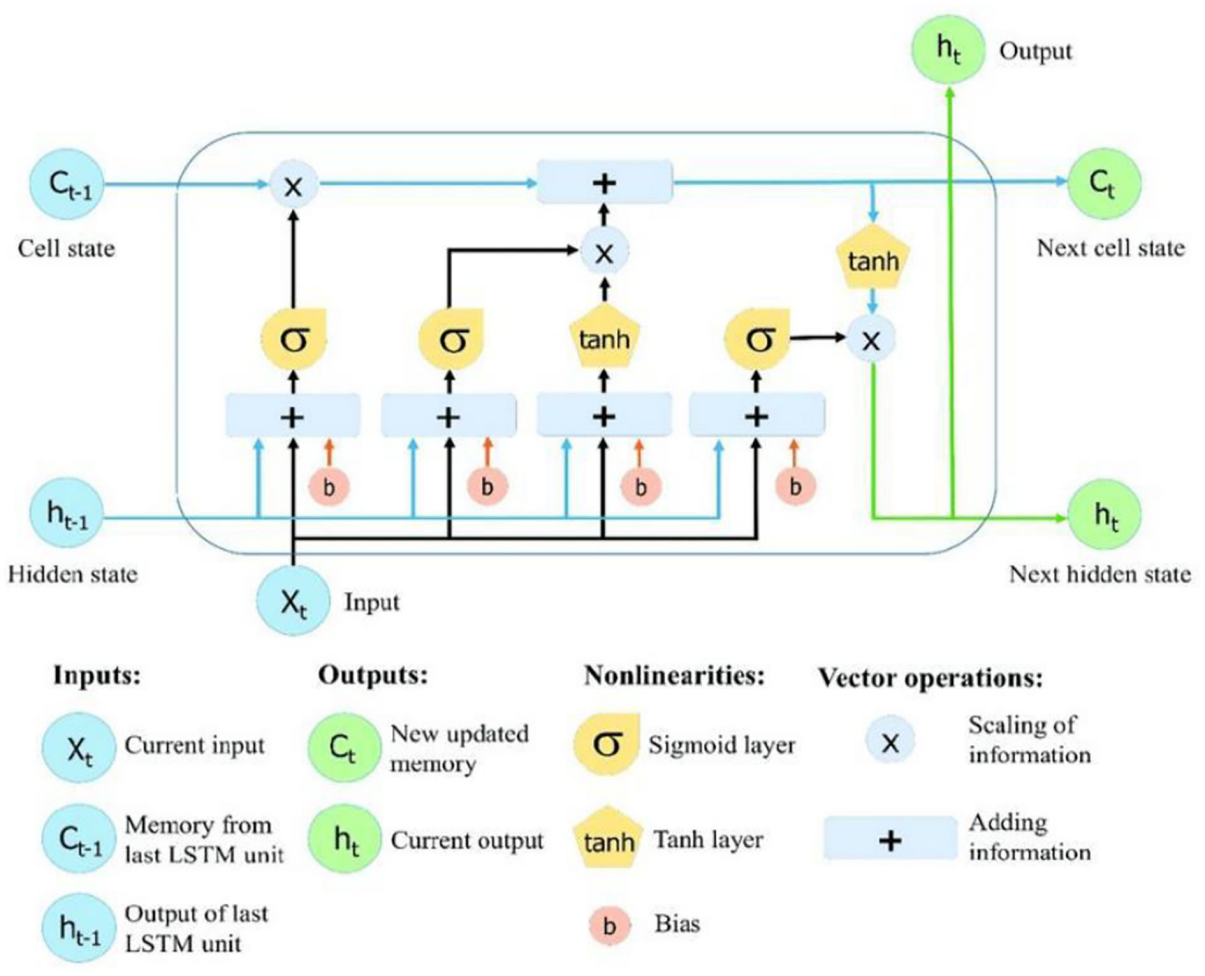
Single LSTM neuron processing data dependencies.

The identification CNN-LSTM activity can be done by introducing the LSTM model on the basis of the CNN model, and it uses CNN to process feature extraction and LSTM to process long-time sequence data. CNN has a two-layered structure: one is the feature extraction layer and its structure adopts the ReLU function as the activation function of the convolutional network, and the other is the feature mapping layer and its structure uses the sigmoid function as the activation function. It takes the pre-processed activity sequence as the input of the model and sets the convolution kernel with a length of *l*. After convolution operation, it gets the new feature sequence with a length of *le* = (*n*−*l*+1), which is shortened through the maximum pooling process, and finally obtains the extracted activity feature vector *v* = (*v*_1_, *v*_2_, …, *v*_*g*_). As the extension of the recurrent neural network, LSTM can better solve the strong data dependency problem in time sequence. The specific model of the LSTM model in activity identification is indicated in Figure 4.

Figure 4 shows the structure of how the single LSTM neuron processes data dependencies. Therefore, LSTM replaces the full-connected layer of CNN and takes the activity feature vector *x* = {*v*_1_, *v*_2_, …, *v*_*g*_} = {*x*_1_, *x*_2_, …, *x*_*g*_} obtained as the LSTM input with each feature vector corresponding to an activity label *A*_*i*_. LSTM determines the reservation of cell state according to the control among its input gate, forgotten gate, and output gate, maintains the relationship between the data with strong dependencies, and solves long-term dependency problems in order to accurately identify the activities. *c*(*t*−1) represents the unit state of the last moment, and the philosophy of LSTM is that the state of the current moment *t* depends on the state of the same neuron in the last moment *t*−1. *h*(*t*) can be defined as the output state of the hidden layer at the current moment, and it can be given by


(8) $$\begin{array}{r} h\left(t\right)\;=\;\log h\left(t-1\right)\;•\;x\left(t\right) \end{array}$$

where *x*(*t*) represents the input data of neuron at moment *t* and *h*(*t*−1) means the LSTM output of the last moment, i.e.,.. The values after forgotten threshold, input threshold, and output threshold can be calculated by:


(9) $$\begin{array}{l} f\left(t\right)\;=\;\sigma \left(W_{\left(f\right)}\;•\;x\left(t\right)+U_{\left(f\right)}•h\left(t-1\right)+b_{\left(f\right)}\right) \end{array}$$

(10) $$\begin{array}{rcl} i\left(t\right)\; & = & \;\sigma \left(W_{\left(i\right)}\;•\;x\left(t\right)\;+\;U_{\left(i\right)}•h\left(t-1\right)+b_{\left(i\right)}\right) \end{array}$$

(11) $$\begin{array}{rcl} o\left(t\right)\; & = & \;\sigma \left(W_{\left(o\right)}•x\left(t\right)+U_{\left(o\right)}•h\left(t-1\right)+b_{\left(o\right)}\right) \end{array}$$

where *f*(*t*), *i*(*t*), and *o*(*t*) represent the output state of the corresponding thresholds, respectively. *W* and *U* are the weights of each branch, and *b* is the bias of each threshold. The activation sigmoid function of input gate decides what information to update, and tanh layer creates a new cell state, which can be given by:


(12) $$\begin{array}{r} \tilde{c}\left(t\right)\;=\;\tanh\left(W\left(c\right)\;•x\;\left(t\right)+U\left(c\right)\;•h\left(t-1\right)\;+\;b\left(c\right)\right) \end{array}$$

Meanwhile, the old cell state will be updated to the new cell state *c*(*t*), which can be given by:


(13) $$\begin{array}{r} c\left(t\right)\;=\;f\left(t\right)\;•\;c\left(t-1\right)\;+\;i\left(t\right)•\tilde{c}\left(t\right) \end{array}$$

The sigmoid function of the output gate determines what information to output and obtains the output *h*(*t*) of the current hidden layer through the tanh function, as suggested by Formula (14):


(14) $$\begin{array}{r} h\left(t\right)\;=\;o\left(t\right)\;•\tanh\left(c\left(t-1\right)\right) \end{array}$$

In the entire LSTM computation process, the output *h*(*t*−1) of the hidden layer of the neuron at moment *t*−1 and *x*(*t*) are the input of the neuron at moment *t*. The input of the neuron in the input layer produces the neuron output *h*(*t*) under the action of activation function, and the memory state *c*(*t*−1) of the neuron cell at moment *t*−1 forms that memory state *c*(*t*) at moment *t*. In the learning process, Formulas (5) and (7) show that the activation function tanh affects *c*(*t*), and the affected *c*(*t*) has point multiplication with the output state *o*(*t*) after the control of the output threshold to get the output *h*(*t*) of the hidden layer at moment *t*. The Softmax activation function normalizes the value of the weight α of the hidden layer, gets the multiplication product and sum of the weight α(*i*) in the hidden layer with the output *h*(*t*) of the hidden layer at the current moment, and obtains the final feature expression *y*(*t*) of the activity identification samples so as to get the respective probabilities of the activities in the sample set, as shown in Formula (15).


(15) $$\begin{array}{r} y\left(t\right)\;=\;\sum \alpha \left(i\right)h\left(t\right) \end{array}$$

Therefore, the interaction among the LSTM input gate, output gate, and forgotten gate decides whether the information should be preserved or abandoned in order to solve the problem of strong data dependencies. This cycle is repeated and after the iteration of one batch (one-time training samples), the accuracy is calculated as the difference between the expected and actual activities.

#### Model structure of automatic sleep stage algorithm

This paper builds the automatic sleep stage algorithm model, mainly including the model structure, size of convolution kernel, and so on. Sleep EEG screens and produces IMF through EMD, and it can effectively present the rhythm wave form and frequency feature of EEG. Therefore, the CNN structure is used to get the time-frequency domain features of each group of IMF, and these features will help with the decision-making of the model on sleep stages and are used to improve the performance of the CNN-LSTM model.

To better simulate the stages of sleep, CNN input includes the EEG and EOG signals which are not processed in the classification stage, and takes a digital matrix as the input. The proposed model in this paper adopts four two-dimensional convolution layers and uses rectified linear unit (ReLU) as the activation function. For each two-dimensional convolution layer, it uses dropout to avoid over-fitting, resulting in neurons being closed or abandoned at a 20% probability. Every convolution layer is followed by one maximum pooling layer before two full-connected layers. The first connected layer has 4,096 cells, and the second one has 1,500 cells. Both layers also adopt dropout to avoid over-fitting and lead to closed or abandoned neurons at a 50% probability. The last is a full-connected layer with a size of 5, and it chooses ReLU as its activation function. All activation functions used in the convolution layers are ReLU with a negative slope of 0.

**Figure 5:**
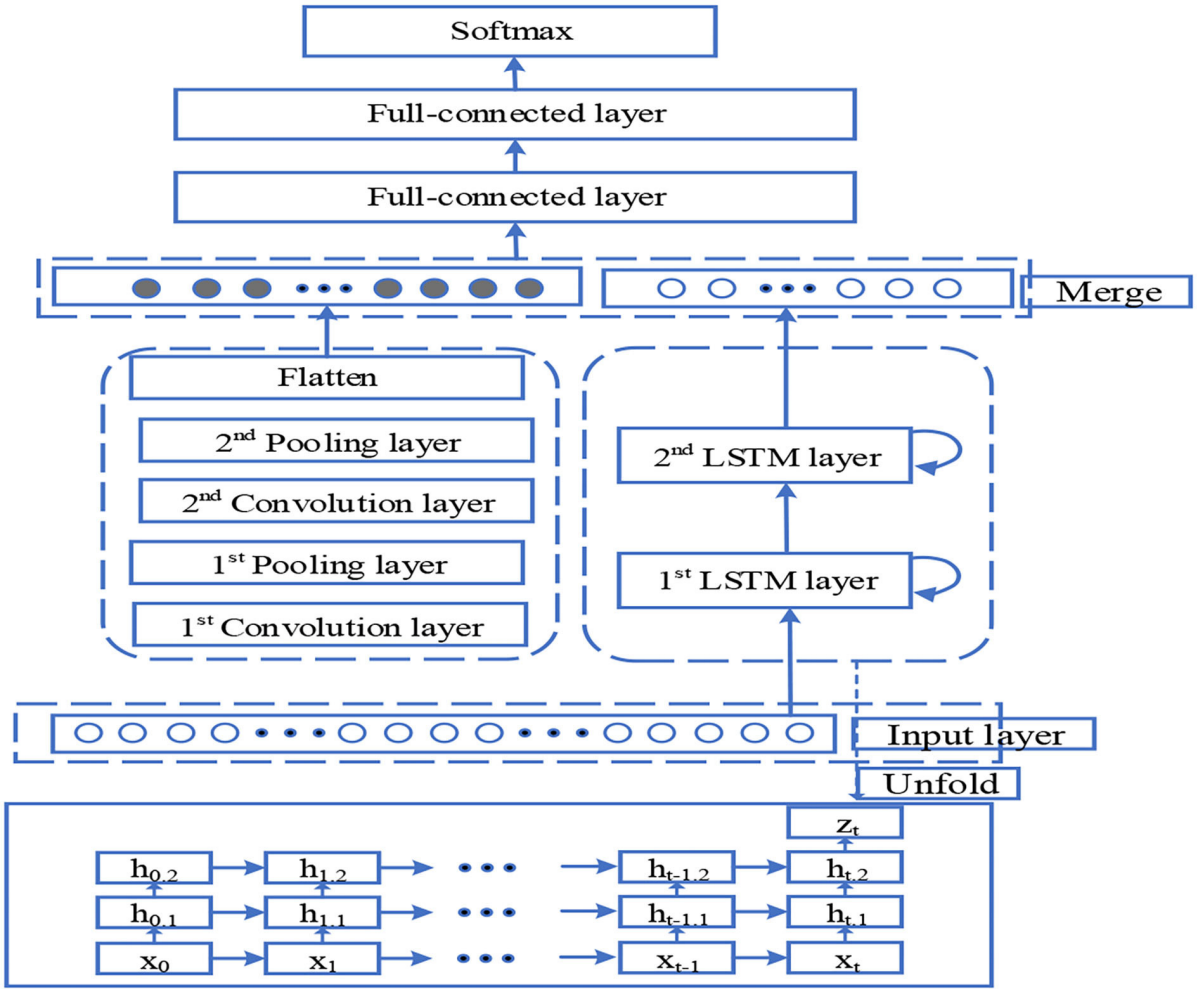
Neural network model of sleep stages.

In EEG-CNN automatic sleep stage model, the input is a 30-s EEG signal with a sampling rate of 200 Hz, and it is represented in a 5 ^*^ 6,000 matrix form. Here 5 means that it adopts the data of five channels, and each EEG sample signal is built alternatively in the order of input layer, Cov, Dropout, Pool until FC1 and FC2, and output. This paper divides EEG and EOG signals of the sleep stages of all types into a test set and training set. To be specific, 20% of all datasets are randomly selected as the test set, and the remaining are selected as the training set in each training stage. When using CNN at a fixed-length time sequence, the output of the convolution directly correlates with the input, the number of convolution layers, and their step lengths. If the output of the last convolution layer is too big, then the majority of the weights will be located in the full-connected layer. CNN connects with LSTM in a parallel manner, and it is used to learn the time sequence features of the output features of two networks. The network output is five sleep stages divided by the AASM sleep staging rule. Figure 5 represents the neural network model of sleep stages.

In Figure 5, the output dimension and core size of each layer are as follows: input layer: 300 × 2; 1st LSTM layer: 300 × 64; 2nd LSTM layer: 64; 1st convolution layer: 296 × 64,5; 1st pooling layer: 98 × 64,3; 2nd convolution layer: 94 × 32,5; 2nd pooling layer: 31 × 32,3; flatten: 992; concatenate: 1,056; 1st full-connected layer: 64; 2nd full-connected layer: 32; and Softmax: 5.

Every convolution layer in this model includes three operations: convolution operation, batch normalization, and activation function non-linearization. Batch normalization acts behind convolution operation, and it is used to re-adjust the distribution of the output in the last layer. It standardizes the input of each hidden layer and prevents the gradient diffusion caused by network deepening. The input layer of the CNN-LSTM designed in this study has 300 ^*^ 2 dimensions. It extracts time sequence features through LSTM, captures profound local correlation information through parallel CNN structure, fuses the convolution features and time sequence features into the full-connected layer, and outputs the multi-classification result from the Softmax layer. The size of batch processing used in this experiment is set to 20. It selects ReLU as the activation function, which is a piecewise linear function and has unilateral inhibition, making the neurons have sparse activation and play the part of automatic decoupling, and increase the convergence speed. The function *f*(*x*) can be given by:


(16) $$\begin{array}{r} f\left(x\right)\;=\;\max\left(0,x\right) \end{array}$$

Then, the full-connected layer maps the high-dimensionality and high-level features obtained from the previous model into the label space with five sleep stages. This model finally uses the Softmax classifier, into which the full-connected layer inputs the five real numbers obtained by multiplying the input feature vector and weight matrix and adding the bias input. The Softmax layer converts it into the probability of the corresponding five sleep stages, and the model judges the staging result according to the maximum probability.

## Contrast experiment and result analysis

### Performance evaluation index

Recall (REC), Precision (PRE), F1 (F1-Score, F1), and ACC (Accuracy) are employed to evaluate the classification performance of the proposed model. ACC is defined as the proportion of the number of samples of the prediction pair to the total number of samples. PRE indicates how many of the predicted positive samples are really positive samples. REC indicates how many positive examples in the sample are correctly predicted. They are common indicators in the statistical analysis. MF1 refers to the mean value of all F1 values in each sleep stage. ACC stands for the percentage of the classification of accurate sleep stages to all sleep stages. REC, PRE, F1, ACC, and MF1 are calculated with the following formulas respectively:


(17) $$\begin{array}{rcl} PRE\; & = & \;\frac{TP}{TP\;+\;FP},\;REC\;=\;\frac{TP}{TP\;+\;FN} \end{array}$$

(18) $$\begin{array}{rcl} F1\; & = & \;2\frac{PRE\;*\;REC}{PRE\;+\;REC} \end{array}$$

(19) $$\begin{array}{rcl} ACC\; & = & \;\frac{\Sigma_{C\;=\;1}^{C}TP_{C}}{N} \end{array}$$

(20) $$\begin{array}{rcl} MF_{1}\; & = & \;\frac{\Sigma_{C\;=\;1}^{C}F1_{C}}{C} \end{array}$$

where *TP*_*c*_ is the number with accurate prediction in all classes and *N* is the total number of samples in all 30 s. *F*1_*c*_ is the score of *F*1 of a certain class, and *C* is the number of sleep stages. *TP* is true positive, *FN* is false negative, and *FP* is false positive.

### Training process

The experiment uses the data of 30 subjects in the MIT-BIH dataset, totaling 60 all-night sleep data. In the experiment, the use of LSTM enables the extraction of the features in a time sequence. In order not to interrupt the time sequence of the sleep stages, a division is made to the data with each subject as the unit. Table 1 presents the division of the subjects in the training set and test set.

**Table 1:** The subject division of training set and test set.

**Data division**	**Training set**	**Test set**
Subject Reference No.	7–30	1–6

The training set is made of the sleep data of 24 people for two nights, totaling 48 nights. The number of samples in each sleep stage in the training set is listed in Table 2.

**Table 2:** Number of 30-s epochs for each sleep stage in training set.

**Sleep stages**	**W**	**N1**	**N2**	**N3**	**REM**
Number	9,563	3,177	17,246	4,854	7,393

The test set comprises the sleep data of two nights of six people, totaling 12 nights. The samples of each sleep stage are presented in Table 3.

**Table 3:** Number of 30-s epochs for each sleep stage in test set.

**Sleep stages**	**W**	**N1**	**N2**	**N3**	**REM**
Number	2,324	1,069	4,835	1,378	1,941

In the training process, evaluate and validate the automatic sleep stage model apart from establishing the test set for the data of six persons, which is totally independent of the training set. Divide the sleep data of 24 persons in the training set into 12 on average, each of which includes the sleep data of four nights. In the 10th round of training, extract and use a ground subset as the validation set.

In the pre-training phase, randomly resample the data in W, N1, N3, and REM stages and perform under-sampled processing in the N2 stage, making the number of samples from six sleep stages every night not to exceed 200 after resampling and under-sampling. The total generations of data training have 100 epochs. A stochastic gradient descent optimizer is used and the initial learning rate is 0.015, which decays 0.1-fold after iterations and training of 20 generations. Set the batch as 10.

### Experiment result and analysis

**Figure 6:**
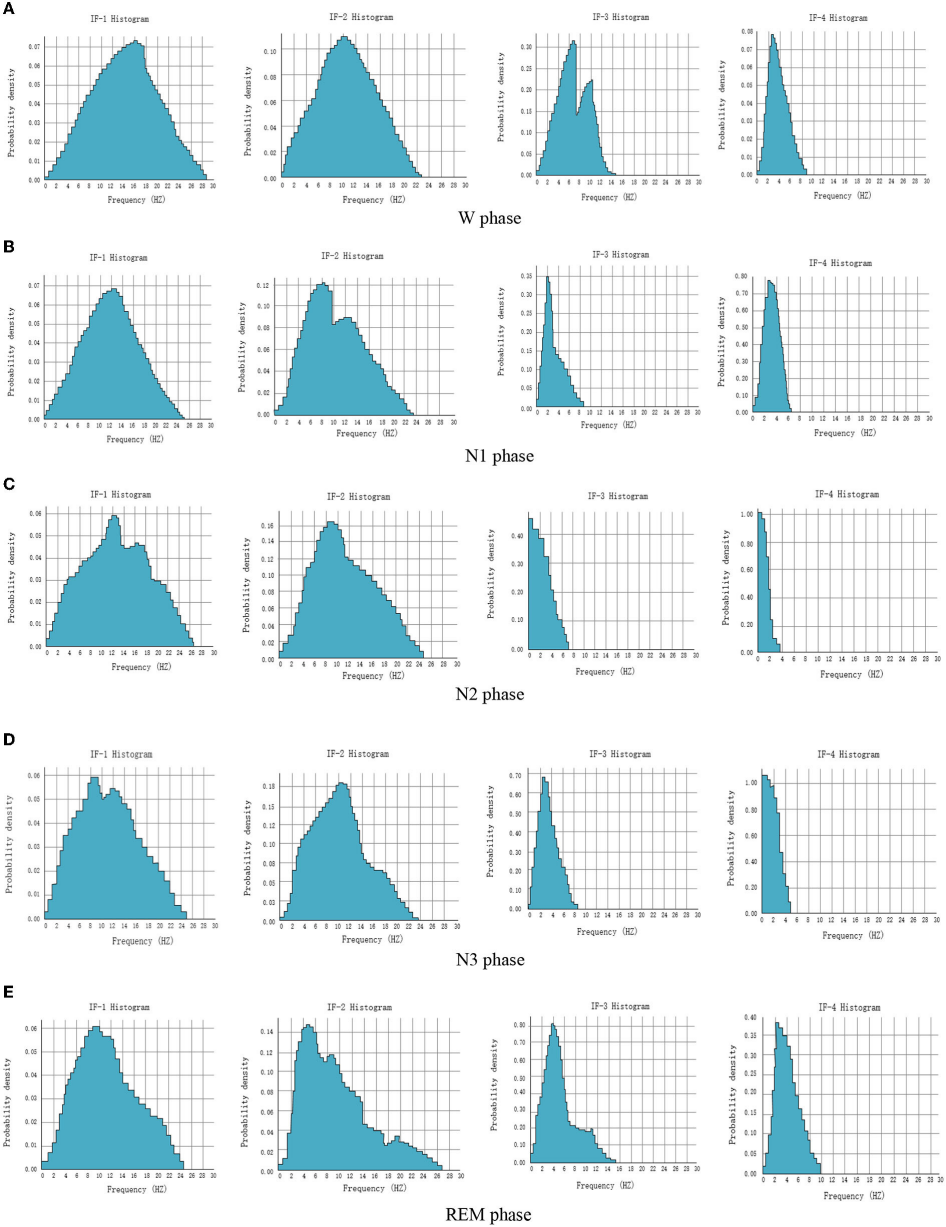
Probability density histogram of instantaneous frequency of EEG signals in each sleep period. **(A)** W phase. **(B)** N1 phase. **(C)** N2 phase. **(D)** N3 phase. **(E)** REM phase.

Hilbert transform focuses on extracting the local characteristics of the signal. HHT amplitude spectrum and marginal spectrum can obtain the time-frequency distribution characteristics of each IMF and the global energy distribution of each frequency. They are distributed in a narrow-band frequency, and the central frequency has a certain distance. It can be seen that the energy of IMF in a local frequency band is relatively concentrated, and the energy proportion of the high-frequency component is relatively small. The multi-resolution characteristics of HHT make the frequency and energy characteristics of this part of the signal more obvious. In order to study the energy distribution characteristics of each sleep period, the data of subjects for 10 days are randomly selected from the MIT-BIH dataset. Forty-five EEG signals are taken from each sleep period every day, and a total of 450 EEG signals are taken during all sleep periods. The instantaneous frequencies of the first four IMF components after empirical mode analysis are calculated, the frequency points are counted, and the probability density histogram is drawn, as shown in Figure 6.

**Figure 7:**
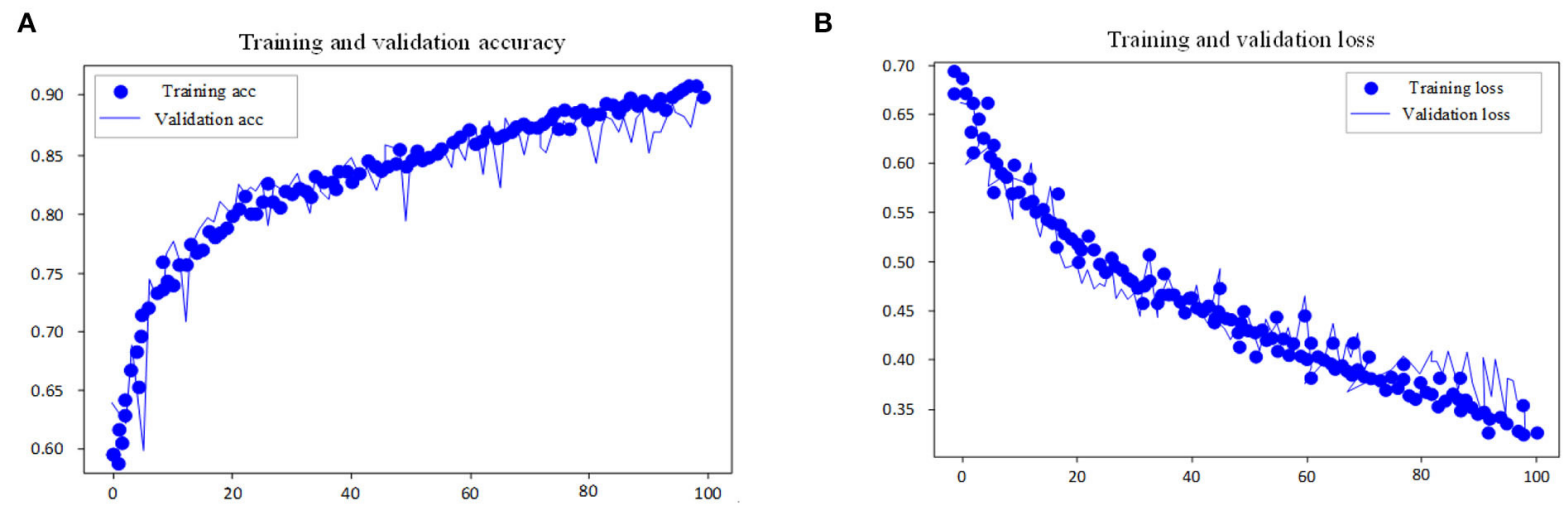
Accuracy and loss curve of pre-training process. **(A)** Training and validation accuracy. **(B)** Training and validation loss.

**Figure 8:**
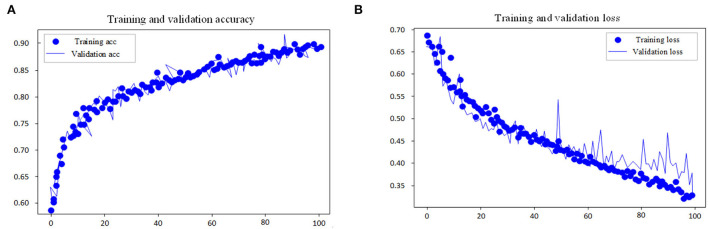
Accuracy and loss curve of fine tuning process. **(A)** Training and validation accuracy. **(B)** Training and validation loss.

This experiment adopts the EGG in MIT-BIH dataset as the input research subject of the model and performs automatic sleep stage model training on the training method and the divided dataset. The goal of the model is to classify sleep into five stages: W, N1, N2, N3, and REM according to AASM rules. In the training steps, the loss and accuracy curves of the training set and test set are shown in Figures 7, 8.

As shown in the performance curves of the training process, the loss function can converge quickly, and the total accuracy reaches 90.6% when the data finishes 100 iterations. But for the test set, its loss function value vibrates greatly. The study shows that with multi-channel EEG and EOG, it is viable to classify the sleep stages with the CNN-LSTM in this study and that EOG data should not be ignored in the study of automatic staging. The training is end-to-end and needs no professional knowledge to select any signal preprocessing method, which is a strength. Neural networks can independently learn the features of each stage and is the most suitable method for classification tasks. From the features of convolutional networks, the convolution layer can learn the excellent filter. Table 4 presents the comparison of performance between the proposed model and other models.

**Table 4:** Performance comparison.

**Author**	**Method**	**F1 scores (%)**					
		**W**	**N1**	**N2**	**N3**	**REM**	**Accuracy (%)**
Fraiwan et al. (2015) (28)	Time-frequency analysis of and random forest classifier	88.32	53.26	85.28	86.31	85.15	84.72
Tsinalis et al. (2016) (29)	Time-frequency analysis and stacked sparse autoencoders	83.47	48.65	85.09	84.28	83.24	81.40
Sors et al. (2018) (30)	Convolutional neural networks	96.86	35.42	89.41	85.52	82.56	89.33
Ours	Multi-channels EEG & EOG CNN-LSTM	90.73	59.78	90.55	83.22	87.38	90.60

It can be seen from Table 4 that the proposed model shows quite good performance and better staging accuracy than other models, which demonstrates the generalization ability of the proposed model. As shown in the results, the multi-channel EEG and EOG model, adopted in this study, is better than the existing multi-channel models. Although other models show excellent performance, the performance of the proposed model is better than other models. From the staging results of different models, it can be concluded that the proposed model has significant advantages.

## Conclusion and future work

In this study, an intelligent automatic sleep staging model based on CNN and LSTM is proposed. As sleep is an extremely complicated process, it is difficult to obtain high accuracy using automatic staging methods (31, 32). The proposed automatic sleep staging method, which is based on EEG, still needs improvement, mainly in the following aspects: (1) The impact of other physiological parameters should be considered. As sleep is a complex process, physiological parameters, such as electrocardio, myoelectricity, electro-oculogram, and breath, have a certain influence on sleep (33). Hence, more parameters should be introduced into the study of sleep stages, which can further increase the accuracy of automatic sleep staging. (2) More feature parameters and experiment samples need to be used to extract more feature parameters (34), including time domain, frequency domain, and non-linear features. Multi-channel data should be selected and more features and data need to be used to make a comprehensive study of sleep stages.

In the future work, we will further explore the application of neural network technology to the automatic sleep staging task, improve the overall accuracy, and provide a theoretical basis for the application of various neural networks to sleep staging.

## Data availability statement

The raw data supporting the conclusions of this article will be made available by the authors, without undue reservation.

## Author contributions

LZ and MD designed the experiments and research project. YZ performed the experiments and analyzed the data. LS wrote the paper. All authors contributed to the article and approved the submitted version.

## Funding

This work was supported in part by the National Science Foundation of China (Grant No. 61772190).

## Conflict of interest

The authors declare that the research was conducted in the absence of any commercial or financial relationships that could be construed as a potential conflict of interest.

## Publisher's note

All claims expressed in this article are solely those of the authors and do not necessarily represent those of their affiliated organizations, or those of the publisher, the editors and the reviewers. Any product that may be evaluated in this article, or claim that may be made by its manufacturer, is not guaranteed or endorsed by the publisher.
